# CIRP contributes to multiple organ damage in acute pancreatitis by increasing endothelial permeability

**DOI:** 10.1038/s42003-025-07772-y

**Published:** 2025-03-10

**Authors:** Wuming Liu, Derek H. Wu, Tao Wang, Mengzhou Wang, Yujia Xu, Yifan Ren, Yi Lyu, Rongqian Wu

**Affiliations:** 1 National Local Joint Engineering Research Center for Precision Surgery and Regenerative Medicine, Shaanxi Provincial Center for Regenerative Medicine and Surgical Engineering, The First Affiliated Hospital of Xi’an Jiaotong University, Xi’an, China; 2 Department of Hepatobiliary Surgery, The First Affiliated Hospital of Xi’an Jiaotong University, Xi’an, China; 3 Macaulay Honors College, CUNY Brooklyn College, Brooklyn, NY USA; 4 Department of Pathology, The First Affiliated Hospital of Wenzhou Medical University, Wenzhou, China; 5 Department of General Surgery, The Second Affiliated Hospital of Xi’an Jiaotong University, Xi’an, China

**Keywords:** Mechanisms of disease, Cell adhesion

## Abstract

Acute pancreatitis can lead to systemic inflammation and multiple organ damage. Increased endothelial permeability is a hallmark of systemic inflammation. Several studies have demonstrated that cold-inducible RNA-binding protein (CIRP) functions as a proinflammatory factor in various diseases. However, its role in endothelial barrier dysfunction during acute pancreatitis remains unknown. To study this, acute pancreatitis was induced by two hourly intraperitoneal injections of 4.0 g/kg l-arginine in wild-type (WT) or CIRP knockout mice. Our results showed that CIRP levels in the pancreas, small intestine, lung, and liver were upregulated at 72 h after the induction of acute pancreatitis in WT mice. CIRP deficiency significantly attenuated tissue injury, edema, and extravasation of Evans blue in the pancreas, small intestine, lung, and liver at 72 h after l-arginine injection. Administration of C23, a specific antagonist of CIRP, at 2 h after the last injection of l-arginine also produced similar protective effects as CIRP knockout in mice. In vitro studies showed that recombinant CIRP caused a significant reduction in transcellular electric resistance in HUVEC monolayers. Immunocytochemical analysis of endothelial cells exposed to CIRP revealed an increased formation of actin stress fibers. VE-cadherin and β-catenin staining showed intercellular gaps were formed in CIRP-stimulated cells. Western blot analysis showed that CIRP induced SRC phosphorylation at TYR416. Exposure to the SRC inhibitor PP2 reduced CIRP-induced endothelial barrier dysfunction in HUVEC monolayers. In conclusion, blocking CIRP mitigates acute pancreatitis-induced multiple organ damage by alleviating endothelial hyperpermeability. Targeting CIRP may be a potential therapeutic option for acute pancreatitis.

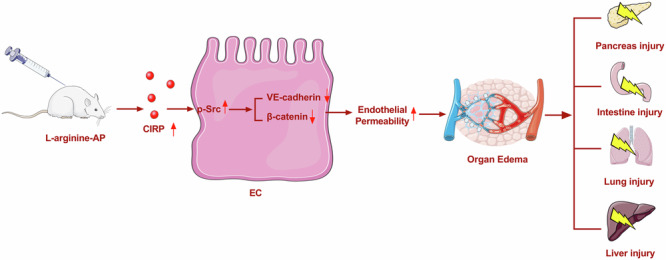

## Abstract (web summary)

CIRP deficiency can significantly ameliorate multiple organ damage in AP by alleviating endothelial cell hyperpermeability and microcirculation disturbance. Targeting CIRP is a potential therapeutic strategy for AP-induced multiple organ damage.

## Introduction

Acute pancreatitis (AP), an inflammatory disease of the pancreatic tissue, is characterized by self-digestion, edema, bleeding, and necrosis caused by the activation of pancreatin in the pancreas by various stimuli^1,2^. The incidence of AP is markedly high worldwide^3,4^. The high mortality rates in patients with severe AP can be attributed to the damaged exocrine pancreas and systemic inflammation caused by the upregulation of cytokine secretion and other toxins in the circulation, resulting in the failure of multiple organs, such as the lung, intestine, central nervous system, and liver^5–8^. The molecular mechanisms underlying AP-associated multiple organ dysfunction have not been elucidated.

Capillary endothelial dysfunction-induced microvascular leakage is a critical event in AP progression^9,10^. This may be related to the release of excessive inflammatory factors and cytokines, resulting in organ capillary endothelial cell damage and increased microvascular permeability, which can lead to organ edema, microcirculation disturbance, and organ failure^10^. In addition to promoting the occurrence and progression of AP, microcirculation disturbance exacerbates organ injury during AP progression^11^. The alleviation of microcirculation disturbance, which is the focus of this study, is a key therapeutic strategy for AP.

CIRP, a critical stress-induced protein, is reported to be involved in several inflammatory responses^12,13^. Additionally, CIRP functions as a damage-associated molecular pattern (DAMP), upregulating the levels of inflammatory factors and chemokines to induce tissue damage^14^. Previous studies have demonstrated that CIRP exerts proinflammatory effects in several pathological conditions, such as hemorrhagic shock, sepsis, and muscle tissue ischemia^12,15^. However, the role of CIRP in AP-induced multiple organ injury has not been previously reported.

C23, an oligopeptide with a high affinity for the CIRP receptor TLR4, suppresses the effects of exogenous CIRP^16^. Extracellular CIRP functions as a DAMP, binding to its receptor TLR4 to induce subsequent inflammatory responses. The C23-mediated inhibition of extracellular CIRP can alleviate acute inflammatory diseases^16,17^. In particular, C23 can alleviate pyroptosis of lung cells and neutrophil extracellular trap (NET) formation in AP^18^. However, previous studies have not determined if the therapeutic effects of C23 on multiple organ damage in AP are mediated through the suppression of exogenous CIRP.

SRC family protein tyrosine kinases are nonreceptor cytoplasmic and membrane-associated protein tyrosine kinases. The SRC signaling pathway plays a critical role in the regulation of both endothelial permeability and proinflammatory mediator-induced transvascular hyperpermeability^19^. Lipopolysaccharide can activate the SRC pathway through TLR4, inducing pulmonary endothelial barrier dysfunction^20^. Previously, we demonstrated that CIRP can induce tissue damage in AP through TLR4, upregulating inflammatory factors^21^. Therefore, the role of CIRP in endothelial cell dysfunction in AP must be examined.

The primary purpose of this study was to explore the role of CIRP in AP-induced multiple organ damage using *CIRP* knockout (KO) mice (*CIRP*^*−/−*^ mice) and exogenous C23 administration.

## Results

### Serum CIRP levels are increased in AP patients

119 patients with AP and 80 healthy controls were involved in this study. Among all 119 AP patients, 55 (46.2%) were diagnosed with mild acute pancreatitis, 36 (30.3%) with moderate-severe acute pancreatitis, and 28 (23.5%) with severe acute pancreatitis. In addition, 50 (42%) were caused by biliary system diseases, 26 (21.8%) were caused by hyperlipidemia, 4 (3.4%) were caused by alcohol, and the remaining 39 (32.8%) were not identified. Severe acute pancreatitis is often accompanied by local complications and organ failure. The local complications associated with acute pancreatitis include pancreatic or peripancreatic fluid collections and pancreatic pseudocysts, abdominal compartment syndrome, intestinal ischemia, gastric outlet dysfunction, splenic or portal vein thrombosis, and pseudoaneurysm^22^. The organ failure induced by acute pancreatitis includes functional impairment of the intestinal, lung, liver, and other organs. We collated the clinical data and found that of all patients, 25 (21%) developed organ failure and 44 (36.9%) developed local complications.

A human cold-induced RNA binding protein (CIRP) ELISA kit was used to measure serum CIRP expression. The mean serum CIRP levels in the control group were 22.9 pg/ml, while that in the AP group was 65.1 pg/ml. Serum CIRP in AP patients was significantly higher than that in healthy controls (Supplementary Fig. 1A). We then compared serum CIRP levels in patients with different degrees of AP. It can be seen that with the aggravation of the disease, the serum CIRP levels of the patients also increased, and the serum CIRP levels in the SAP patients significantly increased (Supplementary Fig. 1B). We analyzed the presence of local complications, and organ failure patients the serum CIRP levels, we found that serum CIRP in the AP patients with complication or organ failure dramatically higher than that patients without (Supplementary Fig. 1C, D).

The SOFA (sepsis-related organ failure assessment) score and APACHE-II (acute physiology and chronic health evaluation) score were often used to quantify organ failure, we analyzed the relationship between these scores and serum CIRP. We found that with the increase of SOFA score and APACHE-II score, the levels of serum CIRP also gradually increased, indicating that serum CIRP level was positively correlated with AP severity (Supplementary Fig. 1E, F).

### CIRP is upregulated in multiple organs of the l-arginine-induced AP model

**Fig. 1:**
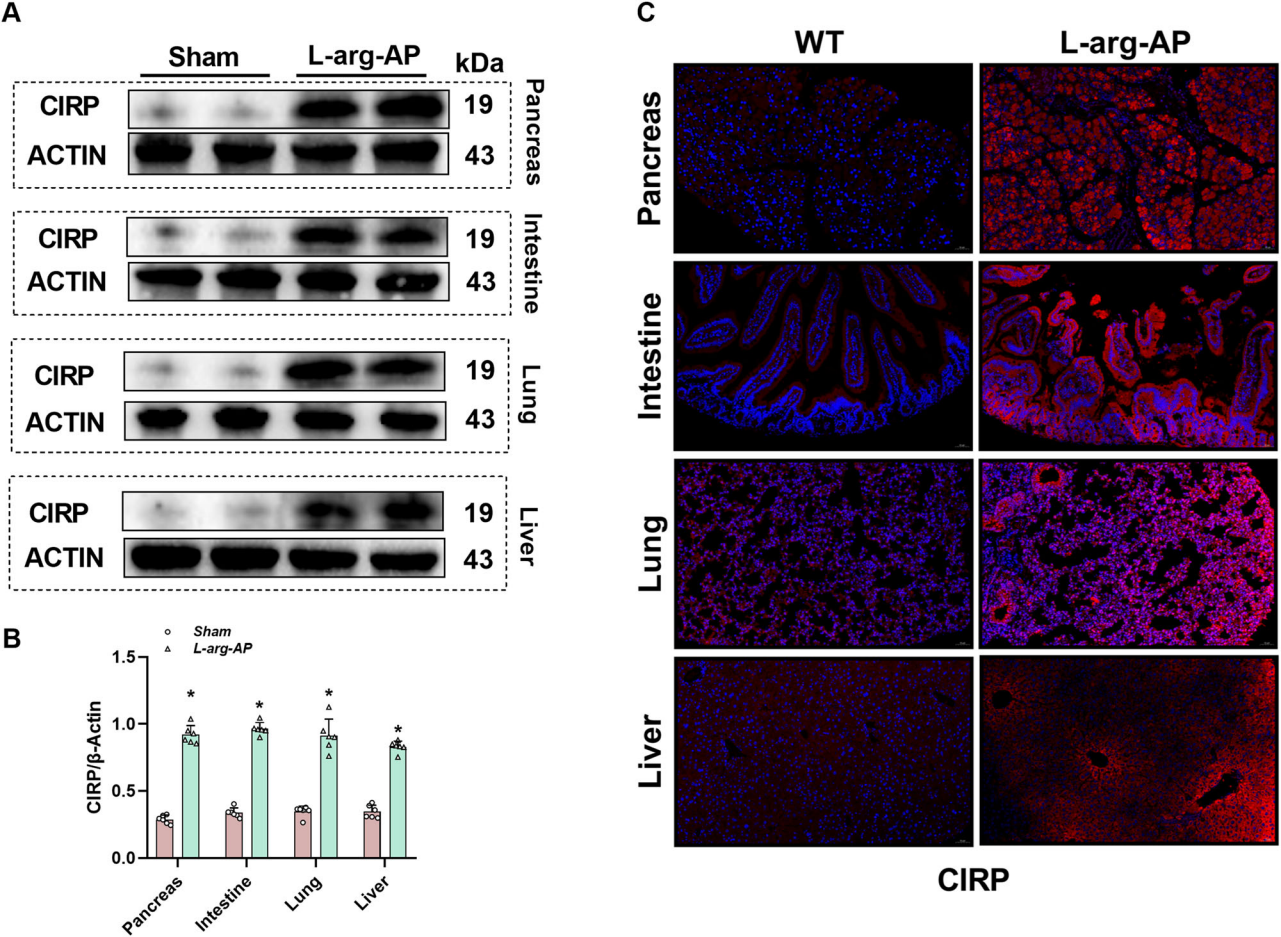
CIRP levels are upregulated in multiple organs in the experimental AP model. In WT mice, l-arginine-AP was induced by two hourly intraperitoneal injections of 4.0 g/kg l-arginine. The animals were sacrificed 72 h after the first injection of l-arginine. **A**, **B** Western blotting analysis of CIRP levels in the pancreas, intestine, lung, and liver tissues of control and l-arginine-treated AP mice. **C** Representative images of immunofluorescence staining of CIRP in the pancreas, intestine, lung, and liver tissues. Scale bar: 50 µm. *n* = 6/group, ^*^p < 0.05 compared with the control group. Data are expressed as mean ± standard deviations.

To explore the correlation between CIRP and multiple organ damage in AP, an experimental AP model was established using l-arginine (4.0 g/kg)^23^. The CIRP levels in the pancreas, intestine, lung, and liver tissues were examined. Western blotting analysis revealed that the CIRP levels were significantly upregulated in the pancreas, intestine, lung, and liver tissues of the l-arginine-induced AP model (Fig. 1A, B). The results of immunofluorescence analysis were consistent with those of western blotting analysis (Fig. 1C).

### CIRP deficiency mitigates pancreatic tissue damage and edema in the AP mouse model

**Fig. 2:**
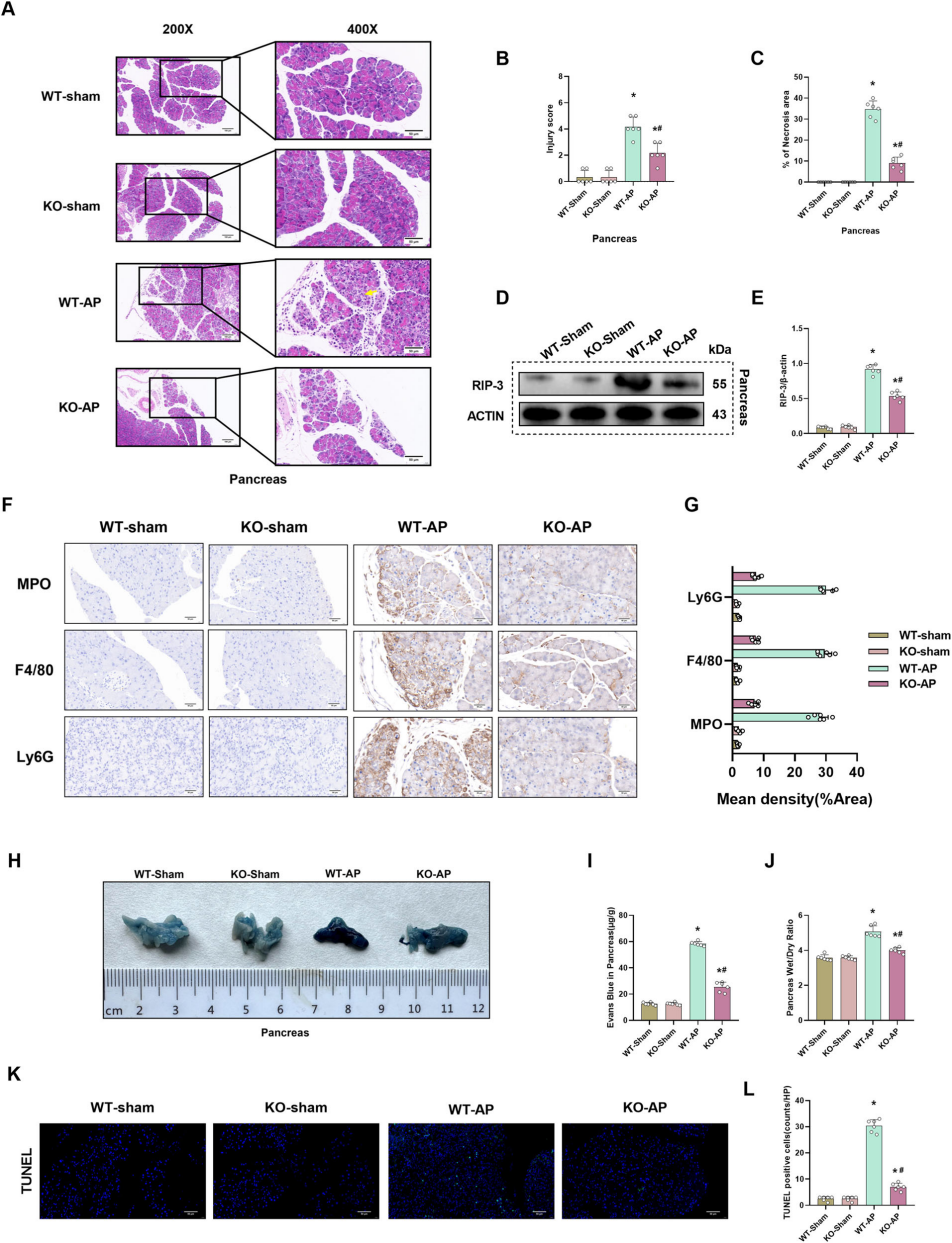
CIRP deficiency mitigates pancreatic tissue damage and edema in the AP mouse model. l-Arginine-AP was induced by two hourly intraperitoneal injections of 4.0 g/kg l-arginine in WT or CIRP KO mice. At 71 h post-first l-arginine injection, Evans blue (EB) dye was intravenously injected into the tail. The animals were sacrificed at 72 h post-first l-arginine injection. The dye was extracted from the pancreatic tissues and quantified. **A** Representative images of hematoxylin and eosin (HE) stained pancreatic sections. Scale bar: 100 µm and 50 µm. **B** Pancreatic injury scores. **C** Quantify pancreatic necrosis area. **D**, **E** Western blotting analysis of the pancreatic levels of RIP-3. **F**, **G** Immunohistochemical analysis of MPO, F4/80, and Ly6G in the pancreas. **H** Representative images of EB dye leakage in the pancreas. **I** Pancreatic EB dye content (µg/g tissue). **J** Pancreatic wet weight/dry weight ratio. **K**, **L** Immunofluorescence analysis of TUNEL (terminal-deoxynucleotidyl transferase mediated nick end labeling). *n* = 6/group; ^*^p < 0.05 compared with the control group; ^#^*p* < 0.05 compared with the WT-AP group. Data are expressed as mean ± standard deviations.

To further explore the role of CIRP in the pathological process of pancreatic injury in AP, l-arginine was used to induce AP in *CIRP*^*−/−*^ mice. Compared with that in WT mice, l-arginine-induced pancreatic injury was significantly alleviated in *CIRP*^*−/−*^ mice (Fig. 2A). Additionally, the pancreatic injury scores and necrosis area were significantly downregulated in l-arginine-treated *CIRP*^*−/−*^ mice (Fig. 2B, C). Furthermore, the pancreatic expression level of RIP-3, a critical necrosis regulator, was downregulated in l-arginine-treated *CIRP*^*−/−*^ mice (Fig. 2D, E). We used immunohistochemical methods to detect inflammatory infiltration and immune cell levels in the pancreas. MPO was used to label inflammatory infiltrates, Ly6G to neutrophils, and F4/80 to macrophages. We found that *CIRP*^*−/−*^ significantly alleviated immune cell expression and inflammatory infiltration in the pancreas, indicating that the level of inflammation in pancreatic tissue was alleviated (Fig. 2F, G). Microvascular permeability of the pancreatic tissue was measured using the Evans blue dye extravasation assay. l-Arginine-induced pancreatic evans blue leakage was significantly mitigated in L-arginine-treated *CIRP*^*−/−*^ mice (Fig. 2H). Additionally, the pancreatic Evans blue dye content was significantly downregulated in l-arginine-treated *CIRP*^*−/−*^ mice (Fig. 2I). These findings indicated that CIRP deficiency alleviates AP-induced endothelial cell hyperpermeability in the pancreatic tissue. Analysis of the pancreatic wet weight/dry weight ratio revealed that CIRP deficiency alleviated AP-induced pancreatic tissue edema (Fig. 2J). Interestingly, immunofluorescent staining results showed that the positive TUNEL cells in pancreatic tissues were also reduced by *CIRP*^*−/−*^, suggesting that CIRP deficiency can alleviate apoptosis in pancreatic tissues (Fig. 2K and L).

### CIRP deficiency mitigates intestinal tissue damage and edema in the AP mouse model

**Fig. 3:**
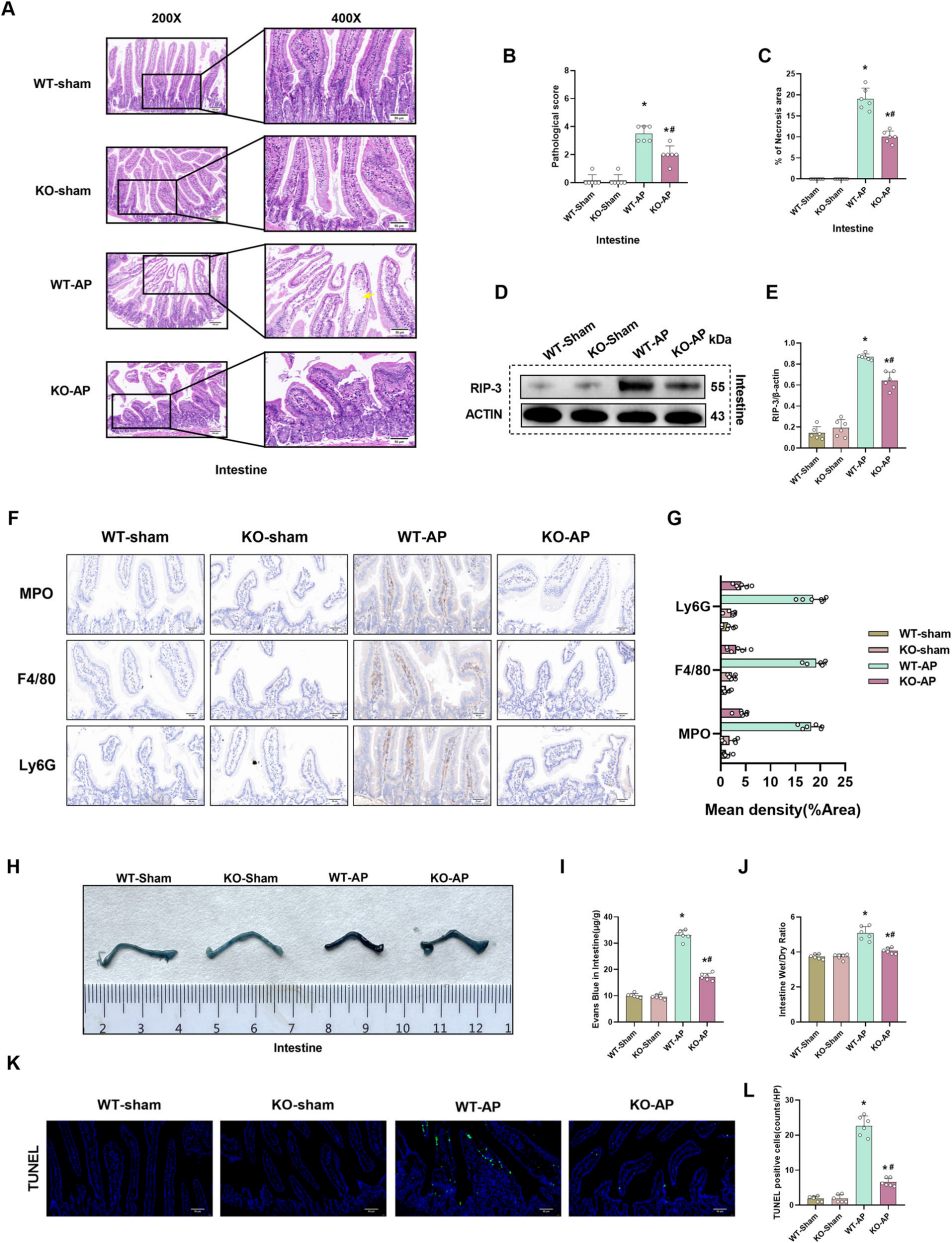
CIRP deficiency mitigates intestinal tissue damage and edema in the AP mouse model. l-Arginine-AP was induced by two hourly intraperitoneal injections of 4.0 g/kg l-arginine in WT or CIRP KO mice. At 71 h post-first l-arginine injection, mice were intravenously administered with Evans blue (EB) dye through the tail. The animals were sacrificed at 72 h post-first l-arginine injection. The dye was extracted from the intestinal tissues and quantified. **A** Representative images of hematoxylin and eosin (HE) stained intestinal sections. Scale bar: 100 µm and 50 µm. **B** Intestinal pathological score. **C** Quantify intestinal necrosis area. **D**, **E** Western blotting analysis of the intestinal levels of RIP-3. **F**, **G** Immunohistochemical analysis of MPO, F4/80, and Ly6G in the intestine. **H** Representative images of EB dye leakage in the intestine. **I** Intestinal EB dye content (µg/g tissue). **J** Intestinal wet weight/dry weight ratio. **K**, **L** Immunofluorescence analysis of TUNEL. *n* = 6/group; ^*^p < 0.05 compared with the control group; ^#^*p* < 0.05 compared with the WT-AP group. Data are expressed as mean ± standard deviations.

Intestinal tissue is susceptible to damage in AP. This leads to the death of intestinal epithelial cells and basement membrane cells and the entry of intestinal bacteria into the blood, further exacerbating systemic inflammatory responses^24^. *CIRP* KO significantly decreased intestinal damage and pathological score in the AP mouse model (Fig. 3A–C). Additionally, *CIRP* KO markedly downregulated the intestinal RIP-3 levels (Fig. 3D, E). Immunohistochemical results showed *CIRP* KO alleviated, inflammatory infiltration and immune cells level in intestinal tissue (Fig. 3F, G). The Evans blue dye leakage assay results revealed that *CIRP* KO significantly mitigated AP-induced intestinal Evans blue dye leakage (Fig. 3H). Analysis of Evans blue dye content demonstrated that *CIRP* KO downregulated the intestinal Evans blue dye content (Fig. 3I). Meanwhile, analysis of the intestinal wet weight/dry weight ratio revealed that *CIRP* KO alleviated intestinal tissue edema (Fig. 3J). *CIRP* KO also significantly mitigated the apoptosis of intestinal tissue (Fig. 3K, L).

### CIRP deficiency mitigates pulmonary tissue damage and edema in the AP mouse model

**Fig. 4:**
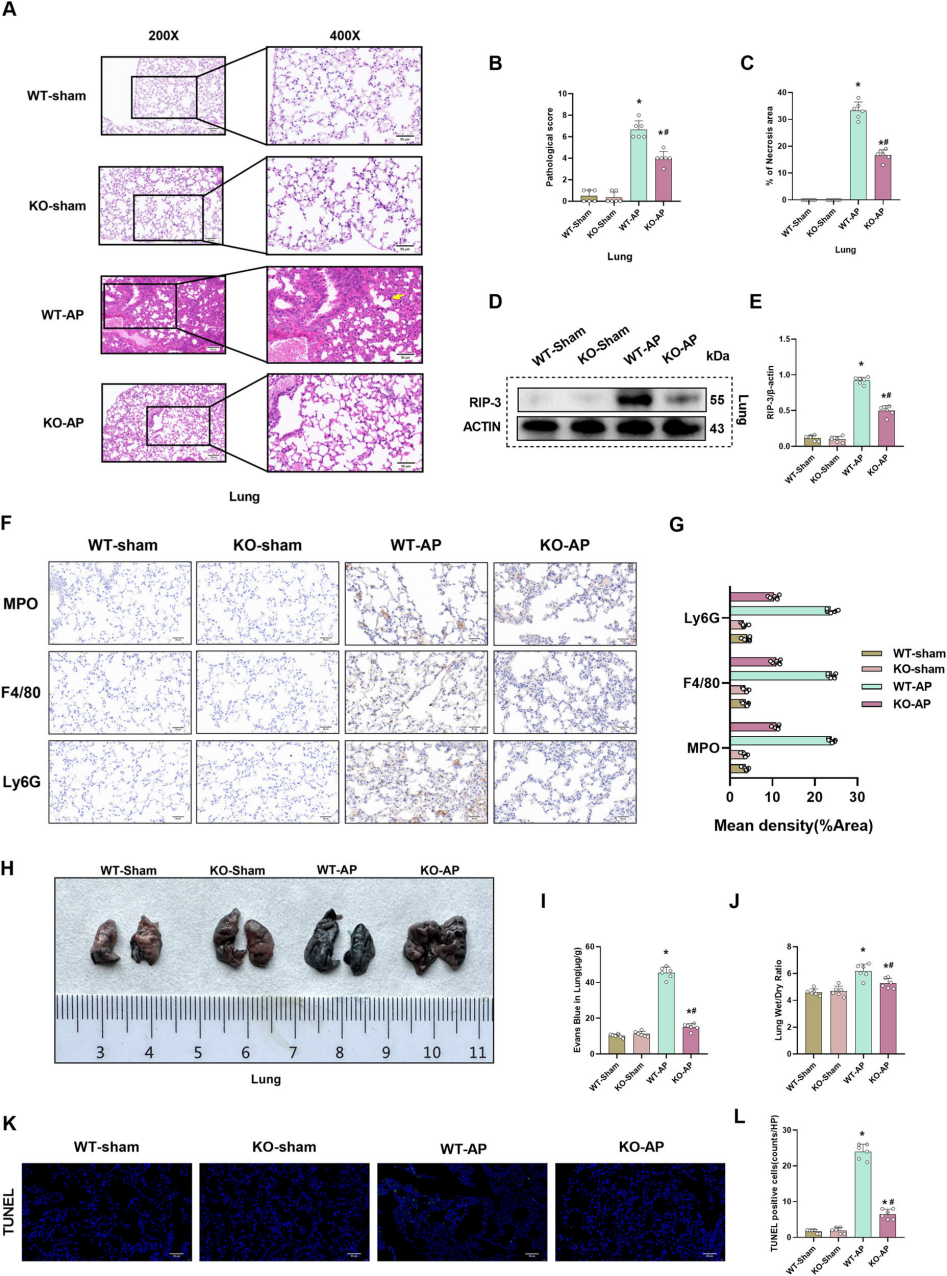
CIRP deficiency mitigates pulmonary tissue damage and edema in the AP mouse model. l-Arginine-AP was induced by two hourly intraperitoneal injections of 4.0 g/kg l-arginine in WT or CIRP KO mice. At 71 h post-first l-arginine injection, mice were intravenously administered with Evans blue (EB) dye through the tail. The animals were sacrificed at 72 h post-first l-arginine injection. The dye was extracted from the pulmonary tissues and quantified. **A** Representative images of hematoxylin and eosin (HE) stained pulmonary sections. Scale bar: 100 µm and 50 µm. **B** Pulmonary pathological score. **C** Quantify pulmonary necrosis area. **D**, **E** Western blotting analysis of the pulmonary levels of RIP-3. **F**, **G** Immunohistochemical analysis of MPO, F4/80, and Ly6G in the lung. **H** Representative images of EB dye leakage in the lung. **I** Pulmonary EB content (µg/g tissue). **J** Pulmonary wet weight/dry weight ratio. **K**, **L** Immunofluorescence analysis of TUNEL. *n* = 6/group; ^*^p < 0.05 compared with the control group; ^#^*p* < 0.05 compared with the WT-AP group. Data are expressed as mean ± standard deviations.

Severe AP is often associated with pulmonary injury. Previous studies have demonstrated that pulmonary edema in AP can cause secondary microvascular leakage and alveolar-capillary barrier disruption^25^. HE staining revealed that the pulmonary tissues of the AP mouse model exhibited a mass of alveolar hemorrhage, inflammatory cell infiltration, and alveolar wall thickening, which were mitigated in *CIRP*^*−/−*^ mice (Fig. 4A). Consistent with these histological changes, *CIRP* KO significantly decreased the pulmonary pathological scores and the pulmonary expression levels of RIP-3 (Fig. 4B–E). These findings indicated that CIRP deficiency mitigates AP-induced pulmonary tissue damage. In addition, *CIRP* KO alleviated inflammatory infiltration and immune cell levels in pulmonary tissue (Fig. 4F, G). Furthermore, Evans blue leakage assay and pulmonary wet weight/dry weight ratio analysis results revealed that *CIRP* KO significantly alleviated endothelial cell hyperpermeability in the lung and pulmonary edema (Fig. 4H–J). Immunofluorescent analysis showed that *CIRP* KO reduced the apoptosis level of pulmonary tissue. (Fig. 4K, L).

### CIRP deficiency mitigates hepatic tissue damage and edema in the AP mouse model

**Fig. 5:**
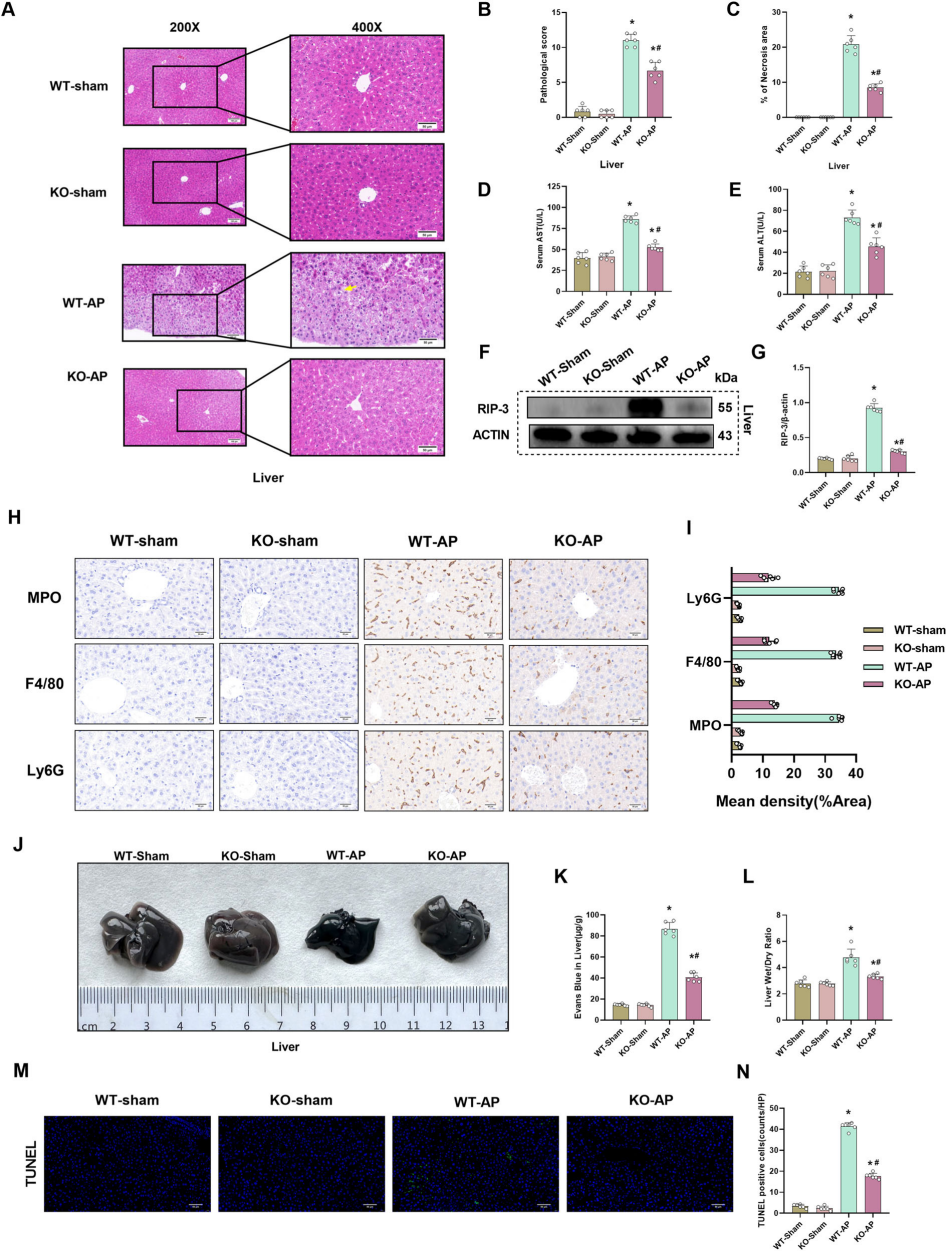
CIRP deficiency mitigates hepatic tissue damage and edema in the AP mouse model. l-Arginine-AP was induced by two hourly intraperitoneal injections of 4.0 g/kg l-arginine in WT or CIRP KO mice. At 71 h post-first l-arginine injection, mice were intravenously administered with Evans blue (EB) dye through the tail. The animals were sacrificed at 72 h post-first l-arginine injection. The dye was extracted from the hepatic tissues and quantified. **A** Representative images of hematoxylin and eosin (HE)-stained hepatic sections. Scale bar: 100 µm and 50 µm. **B** Hepatic pathological score. **C** Quantify hepatic necrosis area. **D** Serum AST levels. **E** Serum ALT levels. **F**, **G** Western blotting analysis of the hepatic levels of RIP-3. **H**, **I** Immunohistochemical analysis of MPO, F4/80, and Ly6G in the liver. **J** Representative images of EB dye leakage in the liver. **K** Hepatic EB dye content (µg/g tissue). **L** Hepatic wet weight/dry weight ratio. **M**, **N** Immunofluorescence analysis of TUNEL. *n* = 6/group; ^*^p < 0.05 compared with the control group; ^#^*p* < 0.05 compared with the WT-AP group. Data are expressed as mean ± standard deviations.

The liver, which is adjacent to the pancreas, is often attacked by the pancreatic contents in AP. Liver injury-induced metabolic and exocrine dysfunctions aggravate the failure of other vital organs, exacerbating organ damage^8^. HE staining revealed that the *CIRP* KO significantly alleviated AP-induced hepatic tissue damage and decreased the hepatic pathological scores, and necrosis areas (Fig. 5A–C). Correspondingly, *CIRP* KO reduced the rise of serum aspartate aminotransferase (AST) and alanine aminotransferase (ALT) induced by AP, which means alleviating damage of hepatic function (Fig. 5D, E). Western blotting analysis demonstrated that *CIRP* KO downregulated the hepatic expression levels of RIP-3 (Fig. 5F, G). Immunohistochemical results showed that *CIRP* KO significantly reduced inflammatory infiltration and immune cell levels in hepatic tissue (Fig. 5H, I). The Evans blue leakage assay results revealed that *CIRP* KO decreased Evans blue dye leakage in the liver (Fig. 5J). Additionally, *CIRP* KO decreased the hepatic Evans blue content and significantly decreased the hepatic wet weight/dry weight ratio (Fig. 5K, L). *CIRP* KO relieved the apoptosis level of hepatic tissue (Fig. 5M, N). These results indicated that *CIRP* KO significantly alleviates AP-induced hepatic endothelial cell hyperpermeability and damage.

### C23 mitigates pancreatic tissue damage and edema in the AP mouse model

**Fig. 6:**
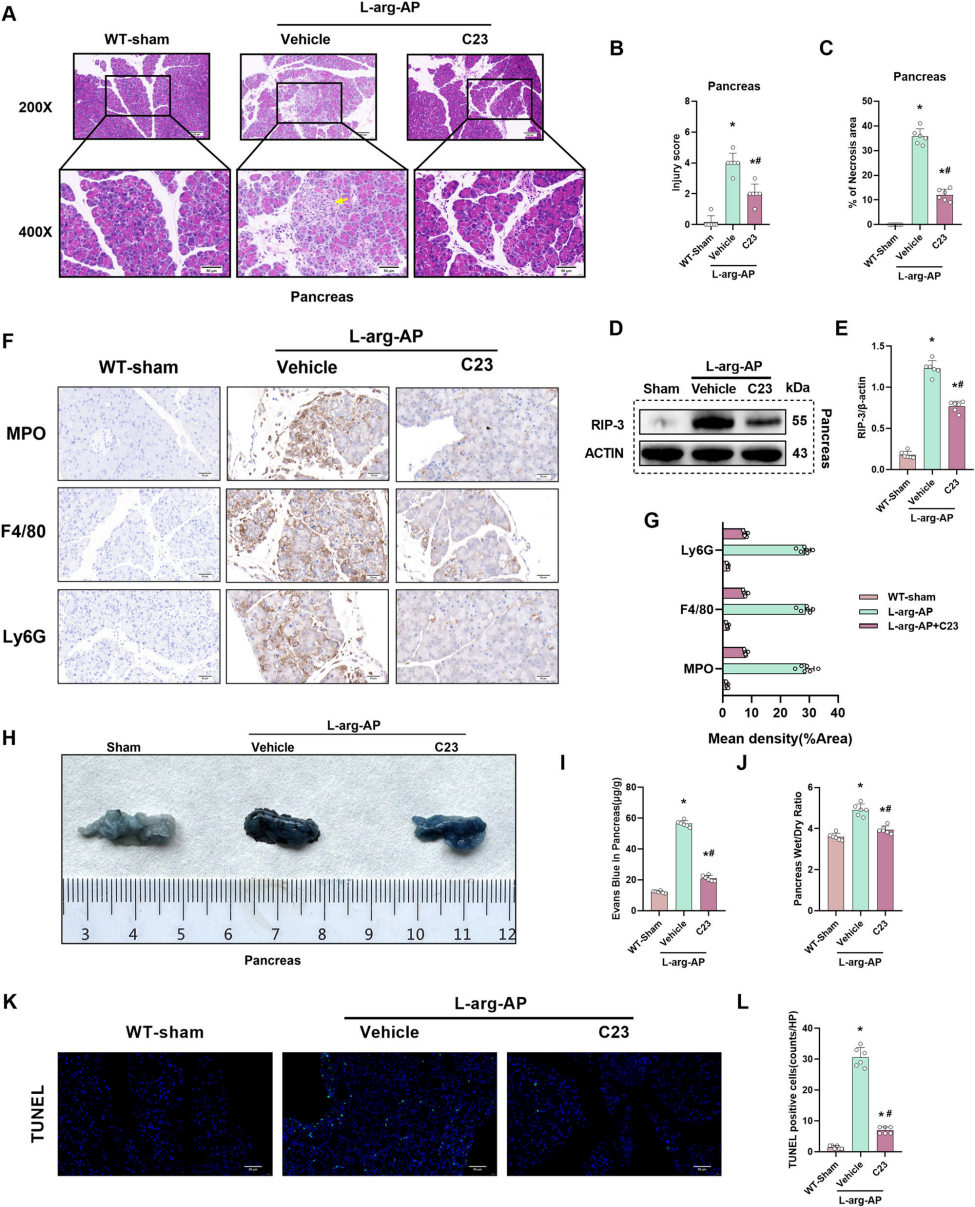
C23 mitigates pancreatic tissue damage and edema in the AP mouse model. In WT mice, l-Arginine-AP was induced by two hourly intraperitoneal injections of 4.0 g/kg l-arginine. At 2 h after the last l-arginine injection, normal saline (vehicle) or C23(8 mg/kg) was administered via intraperitoneal injection. At 71 h post-first l-arginine injection, mice were intravenously administered with Evans blue (EB) dye through the tail. The animals were sacrificed at 72 h post-first l-arginine injection. The dye was extracted from the pancreatic tissues and quantified. **A** Representative images of hematoxylin and eosin (HE) stained pancreatic sections. Scale bar: 100 µm and 50 µm. **B** Pancreatic injury scores. **C** Quantify pancreatic necrosis area. **D**, **E** Western blotting analysis of the pancreatic levels of RIP-3. **F**, **G** Immunohistochemical analysis of MPO, F4/80, and Ly6G in the pancreas. **H** Representative images of EB dye leakage in the pancreas. **I** Pancreatic EB dye content (µg/g tissue). **J** Pancreatic wet weight/dry weight ratio. **K**, **L** Immunofluorescence analysis of TUNEL. *n* = 6/group, ^*^p < 0.05 compared with the control group; ^#^*p* < 0.05 compared with the WT-AP group. Data are expressed as mean ± standard deviations.

This study used C23 (8 mg/kg), an oligopeptide derived from cold-inducible RNA-binding protein, to examine the effect of CIRP inhibition on multiple organ damage in AP. HE staining demonstrated that l-arginine-induced pancreatic injury in the C23-treated group was alleviated when compared with that in the vehicle-treated group (Fig. 6A). Consistently, C23 significantly decreased the pancreatic injury scores and necrosis areas (Fig. 6B, C). Western blotting analysis revealed that the expression levels of RIP-3 in the C23-treated group were significantly lower than those in the vehicle-treated group (Fig. 6D, E). Immunohistochemical results showed that C23 significantly alleviated inflammatory infiltration and immune cell levels in pancreatic tissue (Fig. 6F, G). Evans blue leakage assay and Evans blue content analysis demonstrated that C23 alleviated AP-induced endothelial cell hyperpermeability in the pancreatic tissue (Fig. 6H, I). The pancreatic wet weight/dry weight ratio analysis revealed that C23 alleviated pancreatic tissue edema (Fig. 6J). These results demonstrated that C23 administration alleviates AP-induced pancreatic tissue damage and edema. Similarly, C23 alleviated apoptosis levels of pancreatic tissue (Fig. 6K, L).

### C23 mitigates intestinal tissue damage and edema in the AP mouse model

**Fig. 7:**
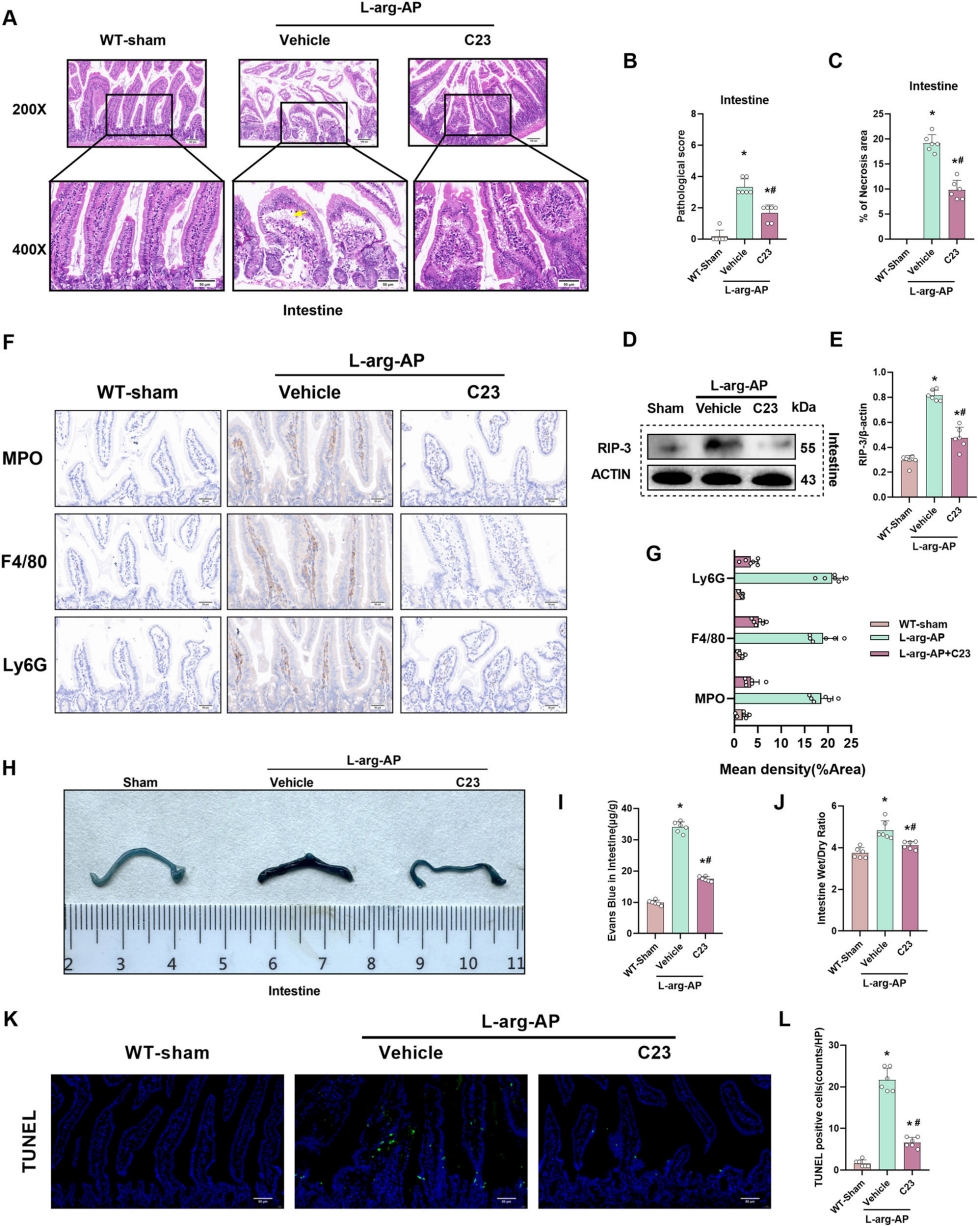
C23 mitigates intestinal tissue damage and edema in the AP mouse model. In WT mice, l-Arginine-AP was induced by two hourly intraperitoneal injections of 4.0 g/kg l-arginine. At 2 h after the last l-arginine injection, normal saline (vehicle) or C23(8 mg/kg) was administered via intraperitoneal injection. At 71 h post-first l-arginine injection, mice were intravenously administered with Evans blue (EB) dye through the tail. The animals were sacrificed at 72 h post-first l-arginine injection. The dye was extracted from the intestinal tissues and quantified. **A** Representative images of hematoxylin and eosin (HE) stained intestinal sections. Scale bar: 100 µm and 50 µm. **B** Intestinal pathological score. **C** Quantify intestinal necrosis area. **D**, **E** Western blotting analysis of the intestinal levels of RIP-3. **F**, **G** Immunohistochemical analysis of MPO, F4/80, and Ly6G in the intestine. **H** Representative images of EB dye leakage in the intestine. **I** Intestinal EB dye content (µg/g tissue). **J** Intestinal wet weight/dry weight ratio. **K**, **L** Immunofluorescence analysis of TUNEL. *n* = 6/group; ^*^p < 0.05 compared with the control group; ^#^*p* < 0.05 compared with the WT-AP group. Data are expressed as mean ± standard deviations.

C23 administration alleviated intestinal damage induced by AP (Fig. 7A–C). Additionally, C23 significantly downregulated the intestinal expression levels of RIP-3 (Fig. 7D, E). C23 decreased the inflammatory infiltration and immune cell level in the immunohistochemical analysis (Fig. 7F, G). Evans blue leakage assay and Evans blue content analysis results demonstrated that C23 alleviated AP-induced endothelial cell hyperpermeability in the intestine tissue (Fig. 7H, I). Analysis of the intestinal wet weight/dry weight ratio revealed that C23 alleviated intestinal edema (Fig. 7J). These results demonstrated that C23 alleviates AP-induced intestinal tissue damage and edema. Immunofluorescent analysis showed that C23 reduced the TUNEL-positive cells, means alleviating the apoptosis of intestinal tissue (Fig. 7K, L).

### C23 mitigates pulmonary tissue damage and edema in the AP mouse model

**Fig. 8:**
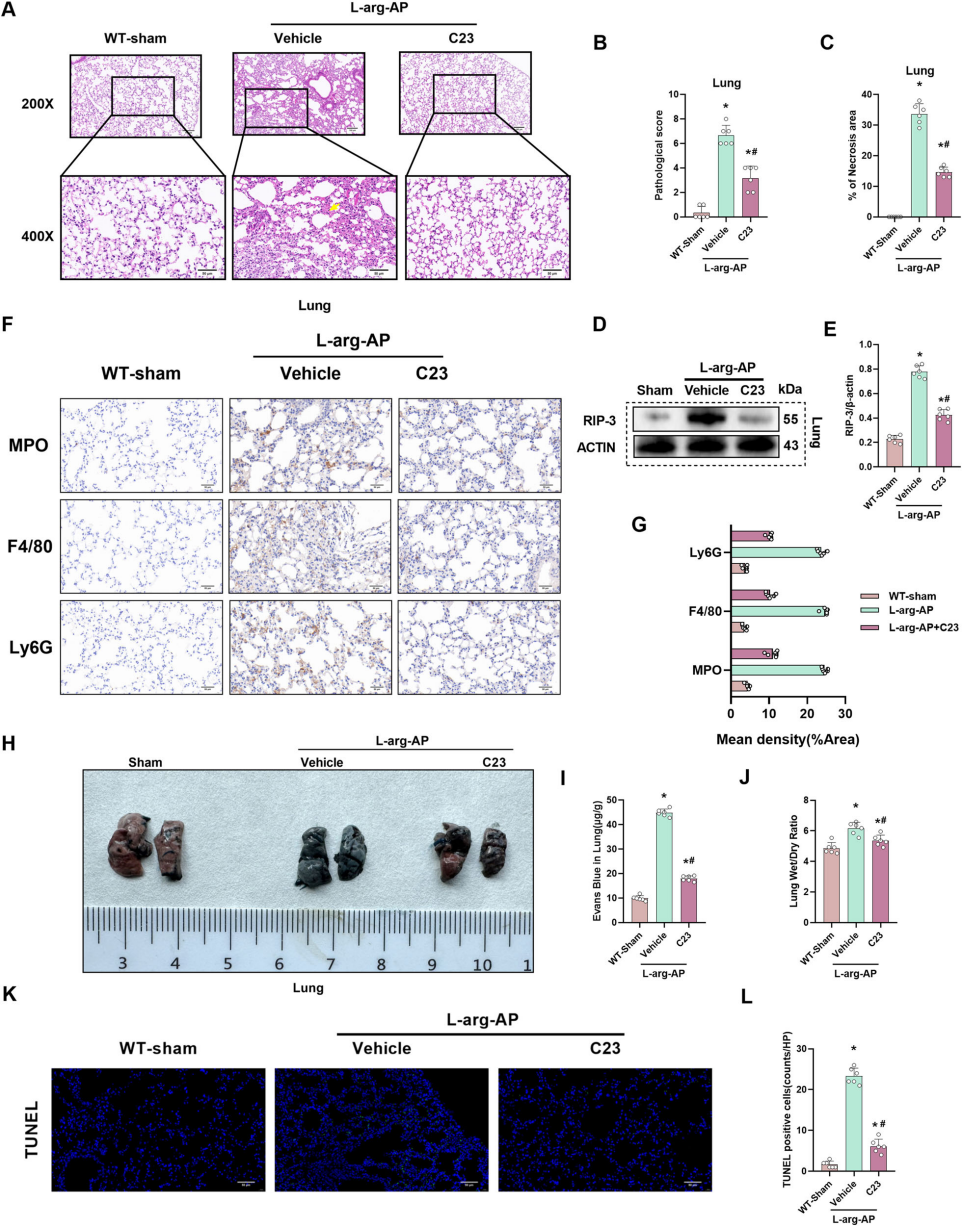
C23 mitigates pulmonary tissue damage and edema in the AP mouse model. In WT mice, l-arginine-AP was induced by two hourly intraperitoneal injections of 4.0 g/kg l-arginine. At 2 h after the last l-arginine injection, normal saline (vehicle) or C23(8 mg/kg) was administered via intraperitoneal injection. At 71 h post-first l-arginine injection, mice were intravenously administered with Evans blue (EB) dye through the tail. The animals were sacrificed at 72 h post-first l-arginine injection. The dye was extracted from the pulmonary tissues and quantified. **A** Representative images of hematoxylin and eosin (HE) stained pulmonary sections. Scale bar: 100 µm and 50 µm. **B** Pulmonary pathological score. **C** Quantify pulmonary necrosis area. **D**, **E** Western blotting analysis of the pulmonary levels of RIP-3. **F**, **G** Immunohistochemical analysis of MPO, F4/80, and Ly6G in the lung. **H** Representative images of EB dye leakage in the lung. **I** Pulmonary EB content (µg/g tissue). **J** Pulmonary wet weight/dry weight ratio. **K**, **L** Immunofluorescence analysis of TUNEL. *n* = 6/group; ^*^p < 0.05 compared with the control group; ^#^*p* < 0.05 compared with the WT-AP group. Data are expressed as mean ± standard deviations.

C23 was used to examine the effect of CIRP inhibition on pulmonary injury. HE staining and pathological score analysis revealed that C23 significantly suppressed AP-induced pulmonary tissue damage (Fig. 8A–C). Additionally, C23 downregulated the pulmonary levels of RIP-3 (Fig. 8D, E). C23 alleviated inflammatory infiltration and immune cell levels in pulmonary tissue (Fig. 8F, G). Furthermore, C23 markedly alleviated endothelial cell hyperpermeability in the lung and pulmonary edema (Fig. 8H–J). The results of immunofluorescent staining indicated that the apoptosis level of pulmonary tissue was significantly alleviated by C23 (Fig. 8K, L).

### C23 mitigates hepatic tissue damage and edema in the AP mouse model

**Fig. 9:**
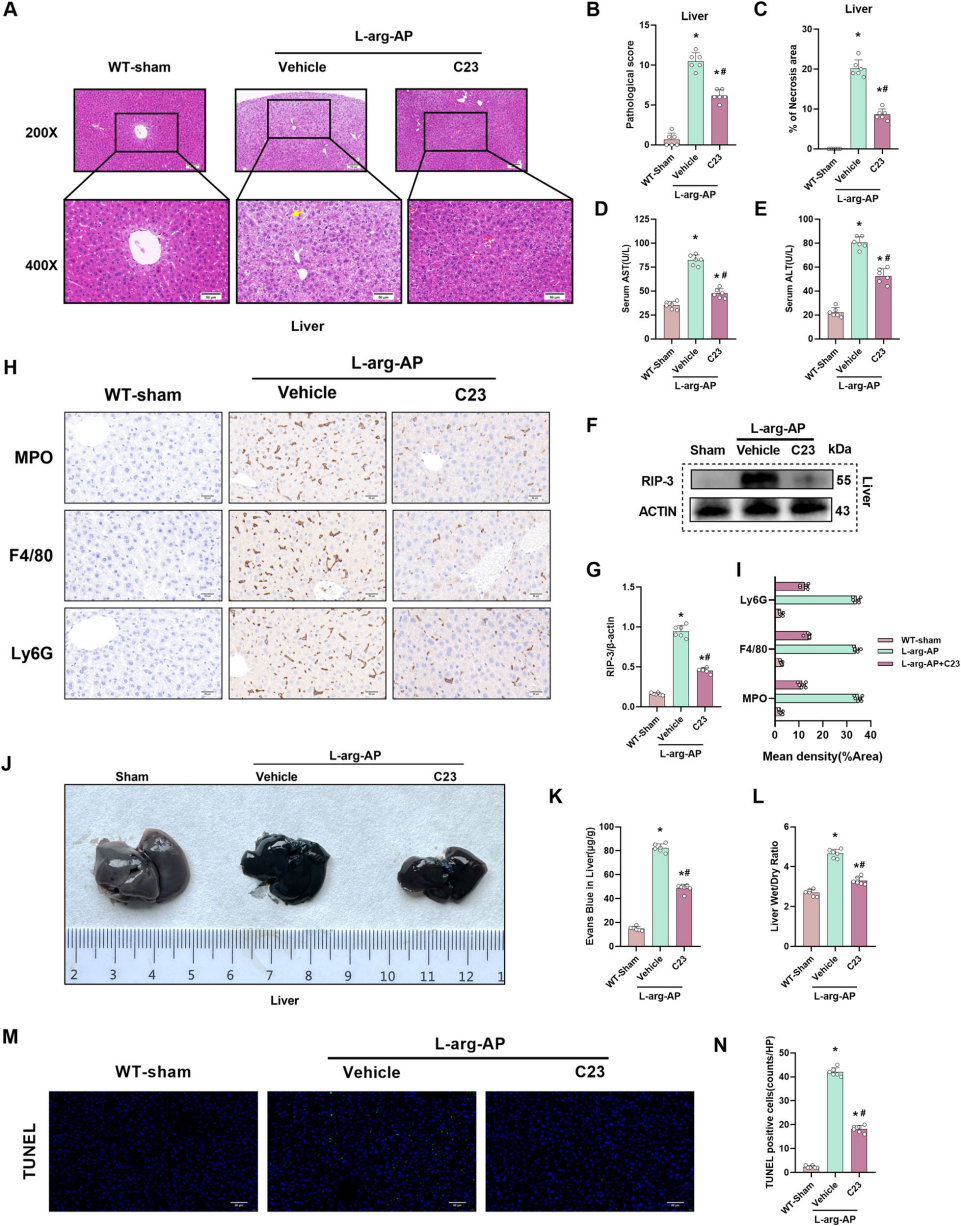
C23 mitigates hepatic tissue damage and edema in the AP mouse model. In WT mice, l-arginine-AP was induced by two hourly intraperitoneal injections of 4.0 g/kg l-arginine. At 2 h after the last l-arginine injection, normal saline (vehicle) or C23(8 mg/kg) was administered via intraperitoneal injection. At 71 h post-first l-arginine injection, mice were intravenously administered with Evans blue (EB) dye through the tail. The animals were sacrificed at 72 h post-first l-arginine injection. The dye was extracted from the hepatic tissues and quantified. **A** Representative images of hematoxylin and eosin (HE)-stained hepatic sections. Scale bar: 100 µm and 50 µm. **B** Hepatic pathological score. **C** Quantify hepatic necrosis area. **D** Serum AST levels. **E** Serum ALT levels. **F**, **G** Western blotting analysis of the hepatic levels of RIP-3. **H**, **I** Immunohistochemical analysis of MPO, F4/80, and Ly6G in the liver. **J** Representative images of EB dye leakage in the liver. **K** Hepatic EB dye content (µg/g tissue). **L** Hepatic wet weight/dry weight ratio. **M**, **N** Immunofluorescence analysis of TUNEL. *n* = 6/group; ^*^p < 0.05 compared with the control group; ^#^*p* < 0.05 compared with the WT-AP group. Data are expressed as mean ± standard deviations.

This study used C23 to examine the effect of CIRP inhibition on AP-induced hepatic tissue damage. C23 ameliorated hepatic tissue damage as evidenced by the alleviation of histopathological changes, downregulation of pathological scores, necrosis areas, and suppression of serum AST and ALT, hepatic RIP-3 level, inflammatory infiltration (Fig. 9A–I). Additionally, C23 decreased the Evans blue dye leakage in the liver and the hepatic wet weight/dry weight ratio, suggesting the alleviation of AP-induced endothelial cell dysfunction in the liver and hepatic edema (Fig. 9J–L). C23 alleviated apoptosis inthe liver (Fig. 9M, N).

### CIRP directly damages the endothelial cell barrier

**Fig. 10:**
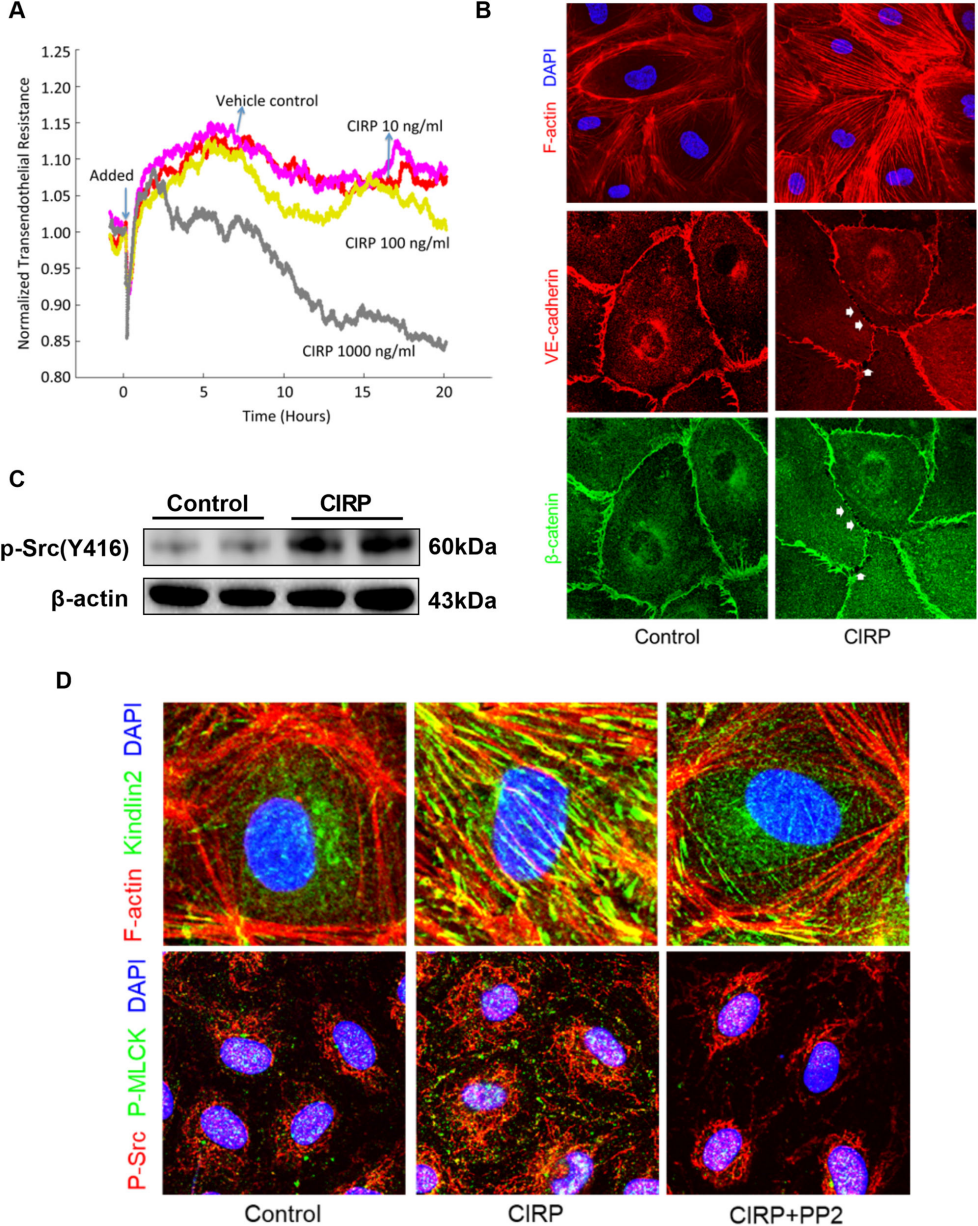
CIRP directly promotes endothelial cell barrier dysfunction. Human umbilical vein endothelial cells (HUVECs) (5 × 10^5^ cells/well) were seeded in a 6-well plate and treated with 1000 ng/mL CIRP in the presence or absence of PP2 (5 µM) for 24 h. **A** Trans-endothelial electrical resistance (TER) in HUVECs. **B** Representative images of immunofluorescence staining of F-actin, VE-cadherin and β-catenin in HUVECs. Arrows in the sections stained for F-actin and VE-cadherin and β-catenin represent gaps between the cells. **C** Western blotting analysis of p-SRC (Y-416) in HUVECs. **D** Representative images of immunofluorescence staining of F-actin, Kindlin-2, p-SRC, and p-MLCK in HUVECs.

To examine the effect of CIRP on endothelial cell permeability, the TER of endothelial cell monolayers was measured. The TER values in the CIRP-treated HUVEC monolayer were lower than those in the vehicle group-treated HUVEC monolayer. CIRP significantly and dose-dependently decreased the TER values with the highest decrease observed in cells treated with 1000 ng/mL CIRP (Fig. 10A). F-actin, Kindlin-2, E-cadherin and β-catenin were stained to assess cytoskeletal remodeling and adherens junction integrity in endothelial cells. It could be seen that CIRP treatment significantly induced the formation of stress fibers (Fig. 10D). Subsequently, the shrinkage of the cytoskeleton leads to an increased intercellular gap, which can be concluded by the apparent reduction of VE-cadherin and β-catenin (Fig. 10B). SRC signaling plays a critical role in the regulation of endothelial permeability and proinflammatory mediator-induced trans-vascular hyperpermeability ^26^. Western blotting analysis revealed that CIRP treatment significantly upregulated the levels of p-SRC (Y416) in HUVECs (Fig. 10C). Previously, we demonstrated that SRC can directly alter the structure of the endothelial barrier by phosphorylation of MLCK, β-catenin, and focal adhesion proteins^27^. Immunofluorescence analysis revealed that CIRP treatment significantly upregulated the expression of p-SRC and MLCK. PP2, an inhibitor of SRC, was used to examine the correlation between CIRP and p-MLCK. Treatment with PP2 markedly mitigated the CIRP-induced upregulation of p-MLCK, this suggested that CIRP promotes MLCK by activating p-SRC (Fig. 10D).

## Discussion

This study demonstrated that the CIRP levels were upregulated in multiple organs of the l-arginine-induced AP mouse model. Additionally, *CIRP* KO or treatment with the CIRP inhibitor C23 significantly alleviated endothelial cell hyperpermeability and organ damage in the AP mouse model. The findings of this study provided potential therapeutic targets for AP-induced multiple organ damage.

Severe AP, which is associated with a high mortality rate, adversely affects the health and quality of life of patients^3,4^. In addition to pancreatic tissue injury and self-digestion, severe AP is associated with the release of several inflammatory factors and cytokines into the blood, which promotes multiple organ damage and exacerbates AP^28^. Previous studies have focused on AP-induced multiple organ dysfunctions, such as intestinal, lung, liver, and central nervous system injuries^5–8^. Intestinal mucosal barrier (IMB) dysfunction is mainly characterized by increased intestinal permeability. A previous study revealed that increased intestinal permeability is the major etiological factor for bacterial and endotoxin translocation during AP progression, contributing to sepsis and multiple organ dysfunction^29^. Targeted alleviation of intestinal injury can significantly mitigate tissue damage in AP^6^. Additionally, AP-associated lung injury manifests as diffuse endothelial injury, leading to the extravasation of macromolecules into the alveolar cavity, pulmonary edema, and even acute respiratory distress syndrome (ARDS)^30^. The liver, which is adjacent to the pancreas, is susceptible to injury. The liver injury-induced metabolic and exocrine dysfunctions aggravate the failure of other vital organs^8^. In this study, CIRP deficiency significantly ameliorated AP-induced organ damage by alleviating endothelial cell hyperpermeability, microcirculation disturbance, and tissue edema.

Severe AP-associated multiple organ damage often causes systemic inflammatory reaction syndrome and even multiple organ dysfunction syndrome^31^. Endothelial barrier dysfunction plays an important role in AP-associated multiple organ dysfunction^28,32^. Changes in endothelial barrier function, including increased cell permeability, release of inflammatory mediators, and expression of cell surface adhesion molecules, are reported to be critical for the pathogenesis of systemic inflammatory response syndromes, multiple organ dysfunction syndromes, and severe AP^28^. Endothelial cell dysfunction can lead to organ microcirculation disturbance^10,33^. The hemorheological changes of microcirculation disorders mainly include microarterial spasm, microvenule congestion, and inflammatory cytokine-induced injury. The alleviation of organ microcirculation disturbance is the focus of AP research and determines treatment efficacy^34^. Previous studies have demonstrated that inflammatory capillary leakage caused by microvascular barrier dysfunction is one of the key steps in the pathogenesis of AP. Thus, the prevention of endothelial dysfunction to reduce capillary permeability is crucial to prevent AP-induced organ damage^10,34^. The results of the Evans blue leakage assay and organ wet weight/dry weight ratio analysis revealed that AP significantly increased the microvascular permeability of the organ, promoting tissue edema and damage. *CIRP* KO or C23 treatment significantly alleviated endothelial cell dysfunction, microcirculation disturbance, and organ edema in AP.

The disruption of endothelial barrier integrity plays a key role in the pathogenesis of various pathological conditions, including ARDS, sepsis, and anaphylaxis^35^. Several mechanisms are involved in the upregulation of endothelial permeability. Some studies have demonstrated that endotoxins can directly cause endothelial cell contraction by activating inflammatory cells, toxic oxygen radicals, peroxides, proteolytic enzymes, and cytokines^36^. In this study, CIRP treatment significantly increased the number of endothelial stress fibers, resulting in cytoskeleton shrinkage and increased intercellular gap. Increased paracellular permeability via intercellular gap formation contributes to vascular leakage.

It is well known that adhesive junctions maintain tight connections between adjacent endothelial cells through adhesin-mediated interactions^19^. VE-cadherin interacts with various intracellular molecules, including β-catenin, plakoglobin (g-catenin), and p120-catenin, which aid in strengthening adhesion^37,38^. CIRP treatment upregulated β-catenin phosphorylation and VE-cadherin separation from the cytoskeleton, weakening the tight connections between endothelial cells. SRC family protein tyrosine kinases are nonreceptor cytoplasmic and membrane-associated protein tyrosine kinases. Under inflammatory conditions, SRC plays an important role in increasing the permeability of endothelial cells^19^. Previous studies have revealed the role of SRC in endothelial cell dysfunction. Hu et al. demonstrated that SRC directly alters the structure of the endothelial barrier by phosphorylating MLCK, β-catenin, and focal adhesion proteins^19^. Paul et al. demonstrated that Src-deficient mice and inhibition of SRC activity can reduce the degree of cerebral edema during stroke^39^. Previously, we demonstrated that irisin enhances endothelial barrier function by suppressing the p-SRC (Y416)/p-MLCK (Y464)/p–β-catenin (Y142) pathway ^27^. In this study, CIRP treatment significantly increased the expression of p-SRC and the phosphorylation of MLCK. PP2, an SRC-specific inhibitor, decreased the expression of phosphorylated MLCK, suggesting that SRC mediates the CIRP activation of MLCK. These results suggest that CIRP adversely affects endothelial cell barrier function by activating the p-SRC/p-MYLKMLCK pathway, promoting endothelial cell hyperpermeability, microcirculation disturbance, and organ edema and injury.

CIRP is involved in the pathogenesis of various inflammatory diseases, such as sepsis and acute lung injury^12,13^. Additionally, CIRP regulates the pathogenesis of several diseases through the classical signal pathway of the TLR4-MD2 complex^40,41^. Previous studies have reported the correlation between CIRP and AP. Linders et al. demonstrated that CIRP can promote tissue injury and AP development by regulating NETs^42^. Xu et al. demonstrated that emodin alleviates severe AP-associated acute lung injury by inhibiting the CIRP-mediated activation of the NLRP3/IL-1β/CXCL1 signaling pathway^18^. The results of this study also showed that the serum CIRP level was significantly increased in AP patients, and was positively correlated with the severity of the disease. The findings of this study were consistent with those of previous studies and suggested that CIRP has a major role in the pathological development of AP-induced multiple organ damage. C23, an oligopeptide extracted from cold-induced RNA-binding proteins, can competitively inhibit CIRP binding to its receptor TLR4. Zhang et al. identified C23 as a potential CIRP antagonist after screening 32 oligopeptides covering the entire sequence of human CIRP for their affinities for the TLR4/MD2 complex^43^. The inhibitory effect of C23 on CIRP has been widely used in various diseases, including sepsis and AP^16,17,44^. In this study, C23 suppressed tissue damage by alleviating endothelial cell dysfunction and microcirculation disturbance in AP through the inhibition of extracellular CIRP. At the same time, we observed that both *CIRP* KO and C23 could alleviate inflammatory mediators and immune cell infiltration in multiple organ tissues, indicating that antagonistic CIRP could improve the level of multiple organ tissue inflammation induced by severe acute pancreatitis.

In conclusion, CIRP deficiency can significantly ameliorate multiple organ damage in AP by alleviating endothelial cell hyperpermeability and microcirculation disturbance. Thus, targeting CIRP is a potential therapeutic strategy for AP-induced multiple organ damage.

## Methods

### Experimental animals and the AP model

Male wild-type (WT) C57BL/6 J mice (Experimental Animal Center of Xi’an Jiaotong University, Xi’an, China) and *CIRP*^*−/−*^ mice (Shanghai Model Organisms Center, Inc., Shanghai, China) aged 8–10 weeks (20–22 g) were housed in the Animal Feeding Center of Xi’an Jiaotong University Health Science Center. *CIRP*^*−/−*^ mice were generated by deleting *CIRP* in WT C57BL/6J mice using clustered regularly interspaced short palindrome repeats (CRISPR)/Cas9 system. The mice were placed in a temperature-controlled room with a 12-h light/dark cycle and fed on a standard laboratory diet. All mice were allowed to fast for 12 h before experimentation. The l-arginine-induced AP model was established as previously described in ref. ^21^. Acute pancreatitis was induced by two hourly intraperitoneal injections of 4.0 g/kg l-arginine (A0013; Solarbio, Beijing, China). Normal saline (vehicle) or C23 (8 mg/kg) was administered 2 h after the last l-arginine injection was administered by intraperitoneal injection. All animal experiments were performed following the guidelines of the China Council on Animal Care and Use and approved by the Institutional Animal Care and Use Committee of the Ethics Committee of Xi’an Jiaotong University Health Science Center, China.

### Cell culture

Human umbilical vein endothelial cells (HUVECs) (CL-0122, Procell, Wuhan, China) were cultured in Ham’s RPMI 1640 medium (PM150110, Procell, Wuhan, China) supplemented with 20% fetal bovine serum (164210-500, Procell, Wuhan, China) in a humidified incubator at 37 °C and 5% CO_2_.

### Clinical data

To explore the expression level of serum CIRP in AP patients, we obtained serum of 119 AP patients and 80 healthy controls from the First Affiliated Hospital of Xi ‘an Jiaotong University between September 2021 and December 2022(ageå 18 years). Sample sizes were based on availability. Basic clinical data of the patients are presented in Supplementary Table 1. This research was carried out according to the code of Ethics of the World Medical Association (Declaration of Helsinki). The study was approved by the Ethics Committee of the First Affiliated Hospital of Xi’an Jiaotong University and all study participants signed the informed consent. The serum CIRP levels were assessed by Human Cold-inducible RNA-binding protein ELISA kit (CSB-EL005440HU, CUSABIO, China).

### Western blotting analysis

Western blotting analysis was performed as previously described in ref. ^45^. The membrane was incubated with primary antibodies (1:1000–2000) at 4 °C overnight, followed by incubation with horseradish peroxidase (HRP)-conjugated secondary antibodies (HRP-conjugated Affinipure goat anti-mouse IgG, SA00001-1, Proteintech, USA and HRP-conjugated Affinipure goat anti-rabbit IgG, SA00001-2, Proteintech, USA) (1:1000) at 37 °C for 45 min. The following primary antibodies were used in this study: anti-β-actin (#3700, CST, USA), anti-CIRP (ab246510, Abcam, USA), anti-RIP-3 (#95702, CST, USA), and anti-p-SRC (GTX24816, Genetex, USA) antibodies. Immunoreactive signals were visualized using a digital gel image analysis system (Bio-Rad, United States). The expression levels of target proteins were calculated using ImageJ software.

### Immunohistochemistry (IHC) assay

IHC analysis was performed as previously described in ref. ^45^. The tissues were fixed in formalin, embedded in paraffin, and then sectioned at 3–5 µm thickness. Subsequently, these sections were deparaffinized, hydrated, and washed with PBS. Then, repairing samples by microwave with 0.01 M citrate antigen retrieval solution and treated with PBS containing 5% H_2_O_2_. Washing with PBS, these sections were blocked in PBS containing 5% serum with 0.3% Triton X-100 for 2 h. Next, they were incubated with anti-MPO (ab208670, 1: 200, Abcam, USA), anti-F4/80 antibody (ab300421, Abcam, USA), and anti-Ly6G antibody (ab25377, Abcam, USA) at 4 °C for 24 h. Then, they were incubated for an hour with the corresponding secondary antibody at room temperature. Further, the samples were subjected to 3,3’-diaminobenzidine color development, hematoxylin staining, alcohol dehydration, cleaning, and gumming. The samples were imaged under a light microscope. For every image, three fields were selected at random. The immunoreactive signals were quantified using ImageJ software.

### Quantitative real-time polymerase chain reaction

Total RNA from tissues was isolated with TRIzol (15596026, Invitrogen, USA) reagent according to the manufacturer’s instructions. The CIRP mRNA expression was normalized to the β-actin. The primers were synthesized by Takara Biomedical Technology as follows: CIRP: 5′-AGCTCGGGAGGGTCCTACAG-3′ and reverse: 5′-GAGGGCTTTTACTCGTTGTGTGT-3′; β-actin: Forward 5′-GGTTCAGGGCGAGGACCATAGAG-3′; Reverse 5′TTTGACAGCGACAAGAAGTGGG-3′. The reaction conditions were performed as previously described in ref. ^45^. The relative levels were calculated using the comparative-Ct method (ΔΔCt method). Each experiment was performed in triplicate, and the data were presented as mean ± standard deviations (SD).

### Histological evaluation of pancreatic, intestinal, pulmonary, and hepatic injuries

At 72 h post-first l-arginine injection, the animals were anesthetized via isoflurane inhalation. The tissues were collected, washed with phosphate-buffered saline, and fixed with 4% formaldehyde for 48 h for embedding.

The histological changes in the pancreas, intestine, lung, and liver were examined using hematoxylin and eosin (HE) staining. The pancreatic pathological score was determined based on Schmidt’s histological scoring system^46^. The intestinal pathological score was calculated following the methods of Zhang et al^47^. The hepatic pathological score (ranging from 0 to 18) was the sum of the scores of the following items: cytoplasmic color fading, vacuolization, nuclear condensation, nuclear fragmentation, nuclear fading, and erythrocyte stasis^48^. The pulmonary pathological score (ranging from 0 to 3) was calculated based on the Osman scoring system, which scores edema, leukocyte infiltration, and hemorrhage^49^. Tissues were randomly selected from each group. Three fields were randomly selected from each group for evaluation.

### Evans blue staining

Microvascular permeability in mice was measured using the Evans blue dye (E2129, Sigma‑Aldrich, USA) extravasation assay. Evans blue was injected into the tail vein at a dose of 20 mg/kg. All mice were anesthetized via isoflurane inhalation and sacrificed after 1 h. The organ tissues were collected, washed with physiological saline, and dried with filter paper. Next, the tissues were incubated with formamide (V900064, Sigma‑Aldrich, USA) (1 mL per 100 mg tissue), homogenized via grinding, and incubated at 60 °C for 12 h. The supernatant was centrifuged at 8000 rpm for 7 min. The quantity of dye extracted was determined at 620 nm by microplate photometer. Results are expressed as mg of dye per g of tissue.

### Tissue wet weight/dry weight ratio analysis

The fresh tissue samples were obtained after the mice were euthanized using isoflurane. The degree of tissue edema was determined by calculating the wet weight/dry weight ratio. The tissues were dried in a 60 °C oven for 48 h. The ratio of wet weight/dry weight was calculated.

### Measurement of trans-endothelial electrical resistance (TER)

The TER of the cell monolayer was measured using an electrical cell-substrate impedance sensing system (Applied Biophysics, USA) as previously described in ref. ^27^. Cell permeability was evaluated by measuring TER across cell monolayers on an 8-well electrode array (8W10E+; Applied Biophysics) using an electrical cell-substrate impedance sensing system (Model 1600R; Applied Biophysics).

### Immunofluorescence staining

Immunofluorescence staining was performed as previously described in ref. ^21^. The tissues were fixed with 4% paraformaldehyde. The primary Anti-beta catenin antibody (ab32572, Abcam, USA, 1:200 dilution), Anti-p-SRC (GTX24816, Genetex, USA); Anti-VE-cadherin (MAB9381, R&Dsystem,USA); Anti-Kindlin-2 (#13562,CST,USA); and Anti-p-MLCK (MBS9386064, Mybiosource,USA) were incubated with samples overnight at 4 °C. Next, the samples were incubated with HRP-conjugated Affinipure goat anti-mouse IgG (H + L) (SA00001-1, Proteintech, China, 1:200) and HRP-conjugated Affinipure goat anti-rabbit IgG (H + L) (SA00001-2, Proteintech, China, 1:200) for 1 h at room temperature. Alexa Fluor™ 594 Phalloidin (A12381, Thermo Fisher Scientific, USA) was used to stain F-actin, following the manufacturer’s instructions. Terminal Deoxynucleotidyl Transferase-Mediated dUTP Nick End Labeling Assay (TUNEL, Roche) was used for the detection of apoptosis cells via immunofluorescence staining. The samples were observed under a confocal microscope (TCS SP8 STED 3X, Leica, Germany). Three fields were randomly selected from each group and imaged under a fluorescence microscope.

### Statistics and reproducibility

All statistical analyses were performed using Prism 8.0 software. Data are expressed as mean ± standard deviations. Means between the groups were compared using the *t*-test or one-way analysis of variance, followed by the Student–Newman–Keuls post-hoc test. Differences were considered significant at *P* < 0.05.

### Reporting summary

Further information on research design is available in the Nature Portfolio Reporting Summary linked to this article.

## Supplementary information

Supplementary Information

Description of Additional Supplementary Files

Supplementary Data1

Reporting Summary

## Data Availability

All data reported in this paper will be shared by the lead contact upon request. Any additional information required to reanalyze the data reported in this paper is available from the lead contact upon request. Numerical source data for all graphs in the manuscript can be found in Supplementary Data 1 file
