# Supplementary material for: CIRP contributes to multiple organ damage in acute pancreatitis by increasing endothelial permeability
Source: Commun Biol. 2025 Mar 10;8:403. doi: 10.1038/s42003-025-07772-y (PMC11894170; doi:10.1038/s42003-025-07772-y)
Supplement: Supplementary file 1 — Supplementary Information [file 42003_2025_7772_MOESM1_ESM.pdf]

Supplementary Materials

CIRP contributes to multiple organ damage in acute pancreatitis by increasing endothelial permeability

Wuming Liu[1,2], Derek H. Wu[3], Tao Wang[1,2], Mengzhou Wang[1,2], Yujia Xu[4], Yifan Ren[1,5], Yi Lyu[1,2], Rongqian Wu[1]

[1] National Local Joint Engineering Research Center for Precision Surgery and Regenerative Medicine, Shaanxi Provincial Center for Regenerative Medicine and Surgical Engineering, The First Affiliated Hospital of Xi'an Jiaotong University, Xi'an, China

[2] Department of Hepatobiliary Surgery, The First Affiliated Hospital of Xi'an Jiaotong University, Xi'an, China

[3] Macaulay Honors College, CUNY Brooklyn College, Brooklyn, NY 11210, USA

[4] Department of Pathology, The First Affiliated Hospital of Wenzhou Medical University, Wenzhou, China

[5] Department of General Surgery, The Second Affiliated Hospital of Xi'an Jiaotong University, Xi'an, China

Supplementary Table 1: Basic clinical data of the patients.

Variables	（%） or Mean ± SD
Total (*N*)	119
Age（years）	52 ± 12.5
Gender（male/female）	71/48
BMI (kg/m^2)	22.4. ± 1.6
Severity of illness	
Mild acute pancreatitis	55 （46.2%）
Moderate severe acute pancreatitis	36 （30.3%）
Severe acute pancreatitis	28 （23.5%）
Epidemic factor	
biliary system diseases	50 （42.0%）
Hyperlipidemia	26 （21.8%）
Alcohol	4 （3.4%）
Others	39 （32.8%）
Treatment	
Supportive treatment	87 （73.1%）
Percutaneous Drainage	12 （10.1%）
Surgery	20 （16.8%）

APACHE II scores	6.33 ± 6.1
SOFA scores	2.62 ± 4
Complication（with/with out）	44/75
Organ failure（with/with out）	25/94
PCT（ng/ml）	2.9 ± 5.8
CRP（mg/L）	139.5 ± 153
Serum lipase（U/L）	1793.5 ± 1831
Serum amylase（U/L）	790 ± 1024
Serum glucose（mmol/L）	8.7 ± 6.6
HbA1c（%）	5.3 ± 3.1
WBC（$\times 10^9$/L）	11.7 ± 5.5
Serum Cr（μmol/L）	78.5 ± 102
Serum BUN（mmol/L）	6.5 ± 8.7
Hct（%）	40.5 ± 9.7
Serum Calcium（mmol/L）	1.95 ± 0.4

APACHE II：Acute Physiology and Chronic Health Evaluation；SOFA: sepsis-related organ failure assessment.

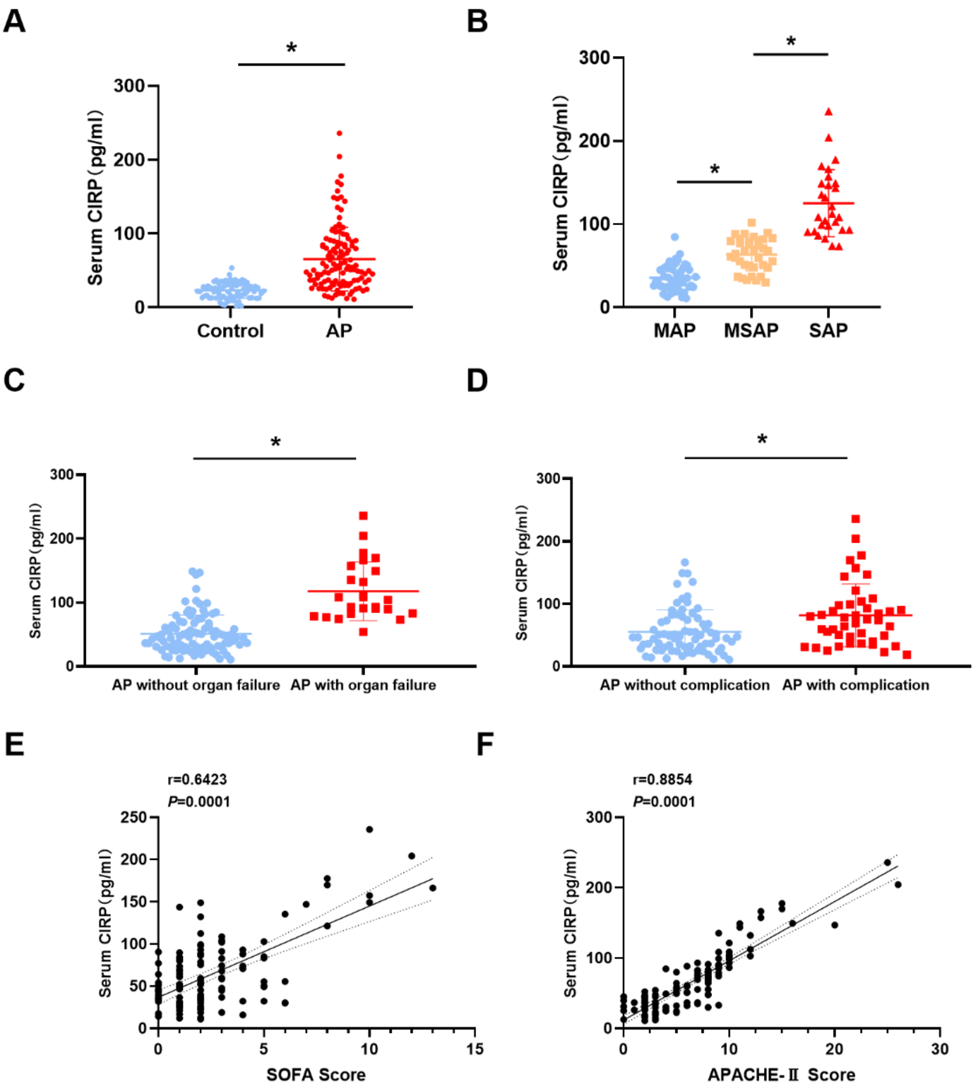

Supplementary Figure 1. Serum CIRP levels are decreased in AP patients.

A. Serum CIRP levels in AP patients and healthy controls.

B. Serum CIRP levels in different degree AP patients. (MAP: Mild acute pancreatitis.; MSAP: Moderately severe acute pancreatitis; SAP: Severe acute pancreatitis.)

C. Serum CIRP levels in AP patients with or without organ failure.

D. Serum CIRP levels in AP patients with or without complication.

E. Correlation analysis of serum CIRP levels and SOFA scores.

F. Correlation analysis of serum CIRP levels and APACHE-II scores.

Data are expressed as mean ± standard deviations. $^{*}p < 0.05$.

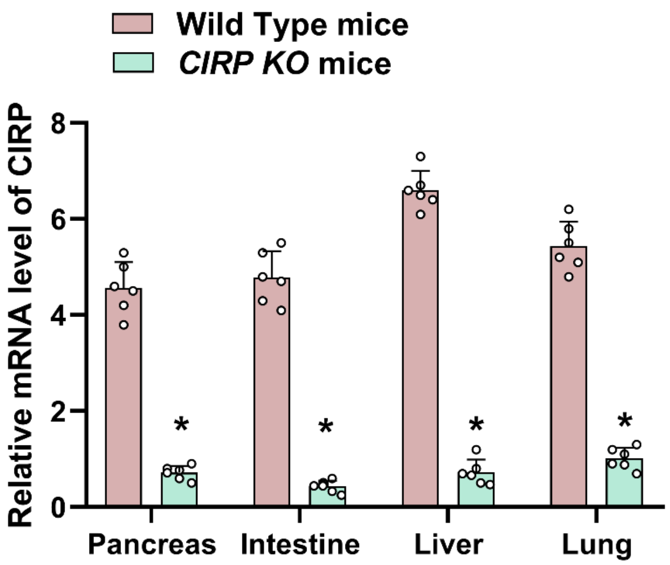

Supplementary Figure 2. The qPCR results of the knockout efficiency of CIRP KO mice.

The CIRP mRNA levels in various organs of *CIRP* KO mice.

n = 6/group; $^{*}p < 0.05$ compared with the Wild Type mice. Data are expressed as mean ± standard deviations.

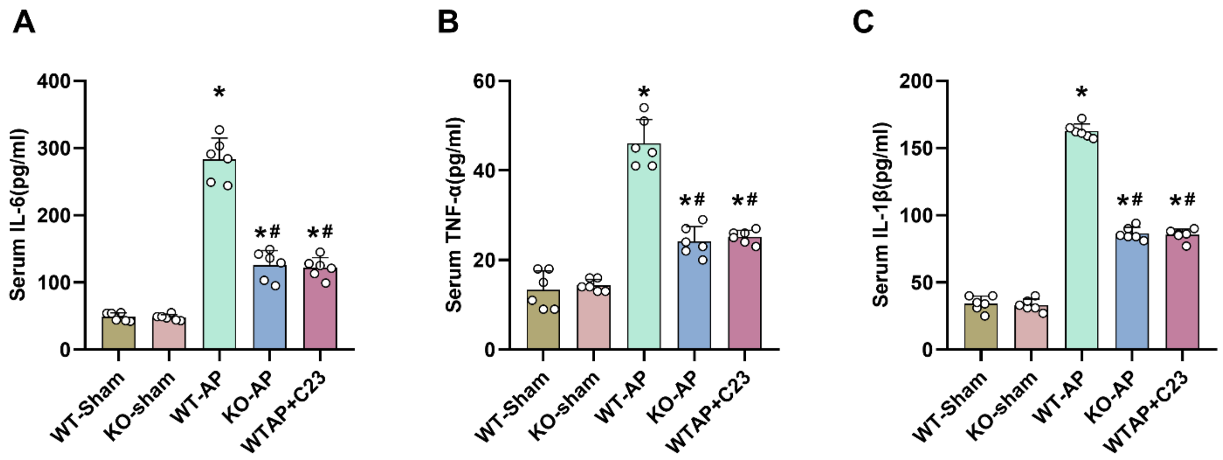

Supplementary Figure 3. The analysis of inflammatory mediators for serum IL-6, TNF-α and IL-1β.

L-Arginine-AP was induced by two hourly intraperitoneal injections of 4.0 g/kg L-arginine in WT or *CIRP* KO mice.

For C23 treatment group, at 2h after the last L-arginine injection, C23(8mg/kg) was administered via intraperitoneal injection. The animals were sacrificed at 72 h post-first L-arginine injection.

A. Serum IL-6 levels.

B. Serum TNF-α levels.

C. Serum IL-1β levels.

n = 6/group; $^{*}p < 0.05$ compared with the sham mice. $^{\#}p < 0.05$ compared with the WT-AP mice. Data are expressed as

mean ± standard deviations.

Supplementary Figure 4. Uncropped and unedited blot images.

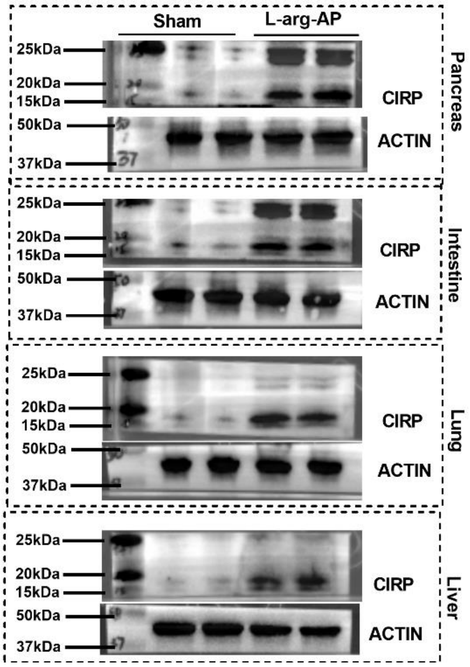

Fig 1A uncropped gel. CIRP levels are upregulated in multiple organs in the experimental AP model.

Entire membranes of the representative Western blot of ACTIN and CIRP, as shown in Fig 1A are provided. Of note, complete membranes were cut horizontally after protein transfer to allow for simultaneous incubation with different antibodies.

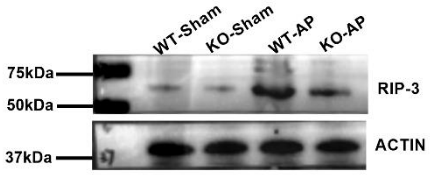

Fig 2D uncropped gel. CIRP deficiency mitigates pancreatic tissue damage and edema in the AP mouse model. Entire membranes of the representative Western blot of ACTIN and RIP-3, as shown in Fig 2D are provided. Of note, complete membranes were cut horizontally after protein transfer to allow for simultaneous incubation with different antibodies.

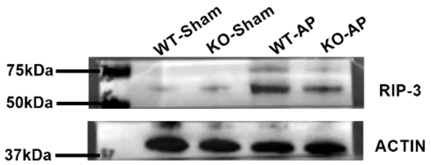

Fig 3D uncropped gel. CIRP deficiency mitigates intestinal tissue damage and edema in the AP mouse model. Entire membranes of the representative Western blot of ACTIN and RIP-3, as shown in Fig 3D are provided. Of note, complete membranes were cut horizontally after protein transfer to allow for simultaneous incubation with different antibodies.

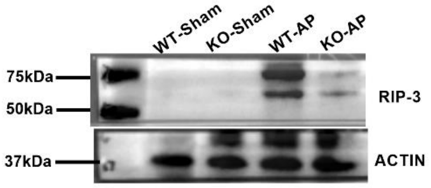

Fig 4D uncropped gel. CIRP deficiency mitigates pulmonary tissue damage and edema in the AP mouse model. Entire membranes of the representative Western blot of ACTIN and RIP-3, as shown in Fig 4D are provided. Of note, complete membranes were cut horizontally after protein transfer to allow for simultaneous incubation with different antibodies.

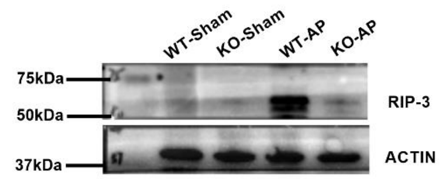

Fig 5D uncropped gel. CIRP deficiency mitigates hepatic tissue damage and edema in the AP mouse model.

Entire membranes of the representative Western blot of ACTIN and RIP-3, as shown in Fig 5D are provided. Of note, complete membranes were cut horizontally after protein transfer to allow for simultaneous incubation with different antibodies.

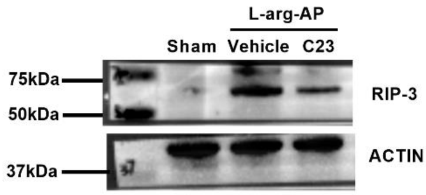

Fig 6D uncropped gel. C23 mitigates pancreatic tissue damage and edema in the AP mouse model. Entire membranes of the representative Western blot of ACTIN and RIP-3, as shown in Fig 6D are provided. Of note, complete membranes were cut horizontally after protein transfer to allow for simultaneous incubation with different antibodies.

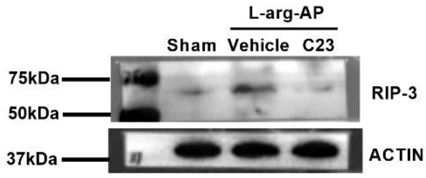

Fig 7D uncropped gel. C23 mitigates intestinal tissue damage and edema in the AP mouse model. Entire membranes of the representative Western blot of ACTIN and RIP-3, as shown in Fig 7D are provided. Of note, complete membranes were cut horizontally after protein transfer to allow for simultaneous incubation with different antibodies.

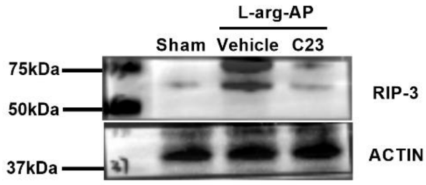

Fig 8D uncropped gel. C23 mitigates pulmonary tissue damage and edema in the AP mouse model. Entire membranes of the representative Western blot of ACTIN and RIP-3, as shown in Fig 8D are provided. Of note, complete membranes were cut horizontally after protein transfer to allow for simultaneous incubation with different antibodies.

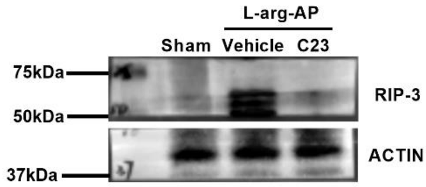

Fig 9D uncropped gel. C23 mitigates hepatic tissue damage and edema in the AP mouse model. Entire membranes of the representative Western blot of ACTIN and RIP-3, as shown in Fig 9D are provided. Of note, complete membranes were cut horizontally after protein transfer to allow for simultaneous incubation with different antibodies.

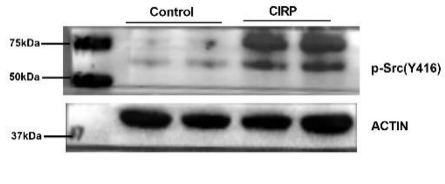

Fig 10C uncropped gel. CIRP can damage the endothelial cell barrier directly. Entire membranes of the representative Western blot of ACTIN and p-Src（Y416）, as shown in Fig 10C are provided. Of note, complete membranes were cut horizontally after protein transfer to allow for simultaneous incubation with different antibodies.
